# Factors associated with the quality of life of people with disabilities living in institutions or attending day care in Luxembourg

**DOI:** 10.1371/journal.pone.0356610

**Published:** 2026-09-08

**Authors:** Marie Blaise, Manon Schroeder, Marc Suhrcke, Germain Weber

**Affiliations:** 1 Luxembourg Institute of Socio-Economic Research (LISER), Esch-Belval Esch-sur-Alzette, Luxembourg; 2 Department of Social Sciences, University of Luxembourg, Esch-sur-Alzette, Luxembourg; 3 Centre for Health Economics, University of York, York, United Kingdom; 4 Faculty of Psychology, University of Vienna, Vienna, Austria; Tribhuvan University Institute of Medicine, NEPAL

## Abstract

**Background:**

To inform policies aimed at improving the quality of life for people with disabilities – an objective enshrined in the United Nations Convention on the Rights of People with Disabilities (UNCRPD) –, there is a need to understand the determining factors of their quality of life.

**Objectives:**

This paper seeks to assess the impact of a uniquely comprehensive set of potential determinants, using a tailor-made survey of people with disabilities in the context of Luxembourg – a country that despite its high level of economic wealth is by some accounts lagging behind in terms of progress towards the UNCRPD.

**Methods:**

We analysed semi-structured interview data from 124 adults with intellectual, motor or autism spectrum disorders living in residential settings or attending day-care services in Luxembourg. Participants were selected from a random sample drawn from 1,115 beneficiaries of Luxembourg’s socio-pedagogical support scheme, stratified by age, gender, residential status and professional status. The data encompasses a range of factors, e.g., socio-economic determinants, social participation, living environment conditions, in addition to various aspects of well-being and quality of life. We employ multiple linear regressions with fixed effects analysis.

**Results:**

Quality of life is significantly associated with the needs for support, living environment settings and – if to a lesser extent – with the frequency of contact with the neighbourhood. By contrast, after adjustment, we did not detect statistically significant independent associations between QoL and disability type, communication skills or mobility limitations.

**Conclusions:**

Our findings lend support to the view that to improve the quality of life of people with disabilities, policymakers should seriously consider focusing on the promotion of greater social participation and independent living.

## Introduction

The concept of ‘Quality of Life’ (QoL) serves as a foundational measure for assessing individual well-being. It adopts a holistic perspective, incorporating the interplay of multiple factors, from physical and mental health, environmental characteristics to social relationships and personal beliefs. According to the World Health Organisation (WHO) [1] QoL reflects an individual’s perception of their life circumstances within the confines of their environment, cultural milieu, personal aspirations, and objectives.

Although the term QoL is not as such mentioned in the United Nations Convention on the Rights of Persons with Disabilities (UNCRPD) [2], its essence permeates throughout its clauses. The core tenets, particularly those highlighted in Article 3, like “Respect for inherent dignity, individual autonomy including the freedom to make one’s own choices, and independence of persons” [2], resonate with the principles intrinsic to enhancing QoL.

People with disabilities face heightened challenges that adversely impact their quality of life [3–6]. Several barriers, ranging from limited educational and employment opportunities to the constraints of higher disability-related expenses and restricted healthcare access, add to the challenges [7]. Hence, tailoring health policies, social practices, and fostering inclusive environments are arguably important to improve the QoL of people with disabilities [8,9].

International research has highlighted various determinants that affect the QoL of people with disabilities, of which health status appears to play a particularly significant role. The detrimental effects of poor mental health [4,10,11], physical health [11], and nutritional imbalances are evident. However, motor and sensory impairments do not seem to considerably impact QoL [12]. The severity of disability has also been shown to play a role: those with profound or multiple disabilities tend to report a lower QoL [6,13–16].

Socio-demographic determinants offer a mixed picture. While some studies indicate gendered differences in QoL, with [17] and [18] showing that men experience better QoL outcomes, [19] found women to be better off and others detected no direct gender effect [15,20]. Similarly, results related to age and marital status are varied. Some studies suggest that age negatively impacts QoL [5,6,17], while others find no such impact [11,15,20].

The WHO emphasises that QoL must be understood in its cultural, social, and environmental context, moving beyond a mere medical perspective. Participation in society, employment status [5,21–23] and involvement in social activities [15,16,24], have been found to significantly promote QoL. Other studies focus on the living environment of people with disabilities, offering mixed results. Living in one’s own larger home has been associated with improved QoL for individuals with disabilities [21], similar to de-institutionalisation and moving to community-based small living arrangements [25,26]. However, studies on living alone yield inconsistent results, with some finding a positive effect (e.g.,), others the opposite [24] and yet others finding no significant link [11]. The impact of the type of living environment, such as day care or residential centre, did not prove as a significant correlate in previous research [12].

Despite a notable body of literature on the determinants of QoL of people with disabilities, important evidence gaps do remain. Most studies focus on a single type of disability, either intellectual [6,26] physical [14,20,21] or consider specific populations, e.g., multiple sclerosis or stroke survivors [13,16,23]. While this has the advantage of targeting specific populations with highly specific needs, it also prevents the result from being generalisable to a broader group of people with disabilities, as addressed by the UNCRPD. In addition, the Luxembourg scenario also offers an interesting – arguably unique – perspective: having ratified the UNCRPD in 2011, Luxembourg introduced subsequent action plans and conventions, such as the ‘Convention Accompagnement Socio-Pédagogique’ (ASP, see S1 Appendix for more details) in 2018, to support people with disabilities [27]. However, criticism persists, especially from the United Nations in 2017, highlighting the country’s comparatively slow progress and lack of comprehensive data [28], despite the economic wealth of the country and its highly developed welfare system.

To our knowledge, no previous study has examined the QoL of people with disabilities in Luxembourg in relation to the combination of factors constituting their living environment, including location and type of residence, type of room, disability status, social interactions, and socio-demographic characteristics. Our study aspires to assess the factors associated with the QoL of people with disabilities in Luxembourg. We aim to adopt a holistic approach, considering socio-demographic characteristics, environmental settings, disability context, and social participation.

## Materials and methods

### Sampling process

The sampling process targeted people with disabilities receiving services under the national scheme for socio-pedagogical support (Convention ASP). Eligible participants were individuals living in community-based residential settings providing 24-hour support or attending day-care services operated by one of the 14 participating service providers governed under the ASP Convention.

Due to data protection regulations and the absence of a comprehensive registry of people with disabilities in Luxembourg, including those covered by the ASP Convention, it was not possible to obtain a complete list of the target population. Instead, the sampling frame was established using administrative data provided by the participating disability service providers, which included all users receiving services under the ASP Convention. In September 2021, a total of 1,115 individuals met the inclusion criteria and constituted the sampling frame.

To ensure that the study sample was representative of the target population, a stratified random sampling strategy was implemented based on four characteristics: age, sex, residential status, and employment status. The final analytical sample consisted of 152 individuals. A weighting procedure was subsequently applied to preserve the distribution of these four characteristics in the target population, thereby ensuring that the analytical sample accurately reflected the demographic and service-related composition of the 1,115 eligible individuals.

### Procedure

For each of the 152 participants, service provider representatives completed a pre-interview. This pre-interview sought to get to know the respondent better, their living environment, their personal and family situation and disability characteristics. It was also intended to find out more about their preferred mode of communication and their capacity to participate in the interview, either on their own or with support, as well as the language spoken, to creating an environment conducive to interaction.

The questionnaire (S2 Appendix) was administered via face-to-face semi-structured interviews at the service providers’ premises. Interviews took place between September, 1 2021 and March, 30 2022. Safety measures concerning the COVID19 crisis were applied [COVID-19 safety measures included wearing masks, limiting the number of people in a room, maintaining physical distance, ensuring proper ventilation by opening windows, and postponing interviews if there was any risk of illness or exposure]. To limit the potential influence of pandemic-related restrictions on responses, participants were explicitly asked to report their social interactions and neighbourhood contacts based on their situation prior to the COVID-19 restrictions. The questionnaire was developed jointly by the multidisciplinary research team, building on previous benchmarks and examples in the literature [9,29,30]. The questionnaire also underwent review by experienced representatives of service providers, operating in the field of disability, and revision after pilot interviews. Two pilot interviews were conducted with people with disabilities who were not included in the study sample but were supported by Convention ASP and received services from the same providers as study participants. All interviewers participated in at least one pilot interview, either as an interviewer or observer, under the supervision of a lead researcher. The pilot interviews were used to train interviewers, test the questionnaire, and assess the feasibility of the Computer-Assisted Personal Interviewing (CAPI) method. Based on the pilot findings, the CAPI approach was replaced by a paper-and-pencil format, and several questionnaire items were reworded to improve clarity and relevance.

In total, five specifically trained interviewers conducted the interviews, including the pilot interviews. Participants could choose to be accompanied by a trustworthy person of their choice. In case of communication difficulties of the participant, supportive communication was used in the form of pictograms, or a trusted person could respond on behalf of the participant. When the participant was not present, a trusted person responded entirely on their behalf (proxy interview). The proxy interviewer was expected to have known the disabled person for at least one year in typical daily situations. All interviews were conducted using paper and pencil and were subsequently entered into a CAPI using the CONVERSO software.

### Ethical considerations

Prior to data collection, the Ethics Committee of the research institute approved the protocol of this study, including the content of the consent form, the questionnaire and the data collection method. All invited participants were then informed about the survey being conducted and their opportunity to participate if they wish. A consent form was sent to them. This document was filled in by the participant himself or, where applicable, by a legal guardian. Moreover, an information letter, also in easy language, was sent to those involved, describing the purpose of the study, the rights of participants and legal guardians, and the use of the data.

### Quality of life

The QoL of individuals with disabilities was assessed using an adapted version of the widely recognised instrument developed by [29]. This model conceptualises QoL across eight key domains: emotional well-being, interpersonal relations, material well-being, personal development, physical well-being, self-determination, social inclusion, and rights. Guided by this framework, we constructed a 16-item scale designed to capture a broad yet concise representation of these domains (see Table 1). In adapting Schalock’s instrument, we did not include the full original questionnaire. Given its length, the shorter version was selected to reduce respondent burden, maintain participant engagement throughout the interview, and minimise the risk of fatigue, incomplete responses, and dropout. The questionnaire also encompassed other important constructs, such as practicability, acceptability, and temporal burden, which required limiting the number of items to prevent respondent fatigue. The 16 items included in the QoL measure were carefully selected to ensure balanced coverage of the eight domains, while maintaining alignment with Schalock’s theoretical model (Table 1). The respondents answered using a 5-point Likert scale, from 1 (completely true) to 5 (not true at all). When needed, the items were recoded, so that the higher the score, the greater the QoL.

**Table 1:** Scale of quality of life.

Items	Adapted scale
My life is very stressful	Excluded
Most of the things that happen to me are good and make me happy	Included
I am happy with my health / I am healthy	Included
There are often arguments and conflicts where I live	Included
I get along well with the people who take care of me	Included
I am considered a stranger	Included
I help a lot with household chores	Included
There are many things I cannot do as well as others	Included
Most of the time I can use my (pocket) money as I want	Included
I decide for myself what I want to do each day (e.g., what time I go to bed, what I want to eat, when my friends visit)	Included
My caregivers (parents) often decide what I should do	Included
What is important to me in my life is often not respected/supported	Excluded
I like to be part of a club (sports, music, church) and I am an active member	Included
Being able to talk to my neighbours is very important to me	Included
I feel rejected by many people in our village (city, neighbourhood)	Included
The way people treat me is not right, not fair	Included

To ensure that participants were able to understand and respond to the items, several measures were implemented: interviewers received specific training on conducting interviews with people with disabilities, questions were reformulated when necessary, using simpler language, and pictograms were made available as a communication support. Where participants were unable to respond themselves, proxy responses were used, an approach also described in QoL research involving people with severe intellectual disabilities when self-report is difficult or not possible [31]. Finally, to enhance response validity, specifically trained interviewers monitored the consistency of responses throughout the interview and checked for potential inconsistencies between items addressing similar domains.

We used an adapted quality of life scale derived from Schalock’s framework. To assess its internal consistency, we calculated Cronbach’s alpha coefficient, a widely used measure of reliability that evaluates the extent to which items within a scale are correlated and consistently measure the same underlying construct. The analysis indicated that two items exhibited low item-total correlations and were not sufficiently consistent with the underlying construct measured by the scale. Consequently, they were removed from the final analysis to improve the scale’s internal consistency, bringing the number of items to 14 (Table 1). Cronbach’s Alpha value of the adapted scale is 0.57.

### Explanatory variables

The factors considered in this study were grouped into four broad categories: socio-demographic characteristics, living environment, disability-related functional limitations, and social interactions. The socio-demographic characteristics included participants’ age, gender, and civil status. The living environment encompassed variables such as place of residence (e.g., city center, outside the city), type of residence (e.g., family home, institutional setting), type of room (e.g., private or shared), and whether the individual attended day care services. Disability-related functional limitations were captured using the ASP disability classification system. The ASP classification covers mild intellectual disability, moderate intellectual disability, severe and profound intellectual disability, poly-disability, motor disability, and autism spectrum disorder. Visual and hearing disabilities fall outside the scope of ASP coverage and are therefore not included in the sample.(See S3 Appendix, Table S3A for the corresponding ICD-11 categories of the ASP classification). Additional indicators included individual capacities in receptive and expressive communication, whether the person had limited mobility, required significant medical care, exhibited behavioural problems, or had objectively identified needs for support. The objective needs for support among individuals with disabilities were assessed using a 14-item scale specifically designed to capture a broad range of support requirements in performing activities such as getting dressed, eating, or going to the supermarket (See Table 2). The respondents answered using a 4-point Likert scale, from 1 (‘I can do it alone’) to 4 (‘someone has to do it for me’). To evaluate the internal consistency of the scale, Cronbach’s alpha was calculated. The resulting coefficient was 0.88, indicating a high level of internal reliability. Finally, the social interactions category included measures such as current employment (e.g., whether the person was working) and the frequency of contact in the neighbourhood.

**Table 2:** Scale of objective needs for support.

Items
Get dressed in the morning
Take a shower or bath
Go to the bathroom
Order something to drink or eat at a bar
Do laundry / Take care of my things
Make a sandwich
Eat myself
Know what is on TV
Make an appointment (e.g., with a doctor) / Arrange a meeting with my friends
Go shopping at the supermarket / go buy candy or toys
Having good relationships with other people / making friends
Communicating personal wishes and desires

### Sample

The selected sample was composed of people with disabilities benefitting from the ASP Convention and included in the initial sample of 152. Minors were excluded (n = 13) from the main analysis because they are still under legal protection of their parents and hence have lower opportunities of self-determination than adults. In addition, children with disabilities have a different living environment compared to adults with disabilities, as well as another perception of their own life as they typically would live in the parents’ home. Out of the 139 adults, we kept those with a minimum response rate of 60% to the QoL items to ensure data quality and to accurately capture the constructs measured by the scale. Robustness tests around this threshold are included in the Results section. These restrictions led to a final working sample of 124 adults.

### Empirical strategy

We seek to identify the main factors associated with the QoL of people with disabilities using an Ordinary Least Squares (OLS) estimator such that:


$$\begin{array}{c} {QoL}_{i}\;=\;\beta_{\text{0}}+\;\beta_{\text{1}}{X\prime }_{i}+\;\beta_{\text{2}}{Z\prime }_{i}+k_{s}+\;\alpha_{p}+\varepsilon_{i} \end{array}$$

where *QoL*_*i*_ represents the quality of life of individual *i*. The vector ***X***_***i***_ includes a comprehensive set of factors such as disability characteristics, living environment settings, social participation, and socio-economic factors. The vector ***Z***_***i***_ contains variables specifically related to interview conditions, such as the language of the interview and whether the respondent attended. $\varepsilon_{i}$ is the error term.

To account for potential variation introduced by different interviewers, interviewer fixed effects $k_{s}$ are included. Additionally, since Luxembourg has 14 service providers operating under the ASP scheme—some of which specialise in supporting individuals with specific disabilities (e.g., autism)—service provider fixed-effects $\alpha_{p}$ are incorporated to control for selection bias related to disability type and provider characteristics.

## Results

### Descriptive statistics

The characteristics of the selected sample – composed of 56 women and 68 men – are presented in Table 3. More than two-thirds were single and aged between 19 and 59. More than 75 percent had intellectual disabilities of varying degree, including autism, almost 6 percent have poly-disabilities and around 15 percent have motor disabilities. This classification of disability is based on categories as defined in the ASP Convention, which we have expanded by adding the autism category as well as the category “others” to capture any other disability situation. In addition, 33 percent had mobility-related limitations and 21 percent reported significant needs due to behavioural problems such as preventing self-harm, tantrums, emotional outbursts and aggression. Almost 16 percent could not express themselves at all during the interview, and a third of the sample could not express themselves sufficiently. The average score of needs for support was 2.08, over a scale from 1 (low support needs) to 4 (high support needs). In terms of living environment, around half lived in a central urban area, in an accommodation managed by a service provider and the vast majority occupied a single room. In terms of social interactions, the distribution was almost equally balanced between daily, weekly or every two- or three-weeks contact, between 15 and 20 percent of the sample, however, almost 37 percent reported having no contact at all with their neighbourhood. Finally, only 27 percent of the sample worked in workshops with a salary under the “disabled worker scheme”. Those non-working were entitled to a state guaranteed minimum monthly living income.

**Table 3:** Descriptive statistics of the sample.

Variable	Freq.	%
**Socio-demographic characteristics** Gender		
Female	56	45.16
Male	68	54.84
Age		
19-59	88	70.97
60+	36	29.03
Marital status		
Single	103	83.06
Married	10	8.06
Divorced	10	8.06
Widowed	1	0.81
**Living environment**		
Institutional living status		
Daycare	29	23.39
24h residential setting	95	76.61
Type of residence		
in independent housing managed by the service provider	10	8.06
in housing within a facility, including only housing owned by a service provider	73	58.87
In private housing with parents	16	12.90
In private accommodation with family members (but not parents)	10	8.06
Others	15	12.10
Localisation of the place of residence		
The place of residence is in an area where the rest of the population also lives, e.g., a residential area, downtown or in the heart of the village	62	50
The place of residence is located on the outskirts of a city (suburb)/village	46	37.10
The place of residence is outside a city/town	16	12.90
The type of the room		
Individual with one single bed	117	94.35
For two persons (separate beds)	2	1.61
For two persons (double bed)	4	3.23
Three or more people (separate beds)	1	0.81
**Disability situation**		
Disability classification (ASP Convention)		
Mild intellectual disability	26	20.97
Moderate intellectual disability	41	33.06
Severe and profound intellectual disability	26	20.97
Poly-disability	7	5.65
Motor disability	18	14.52
Autism	5	4.03
Other	1	0.81
Very limited mobility	41	33.06
Significant medical care needs	3	2.42
Significant needs related to behavioural problems	26	20.97
Level of language skills in the language of the interview: receptive communication		
Sufficient	75	60.48
Limited	38	30.65
None	11	8.87
Level of language skills in the language of the interview: expressive communication		
Sufficient	62	50
Limited	42	33.87
None	20	16.13
Objective needs for support	2.08*	0.66**
**Social interactions**		
Working	34	27.42
Frequency of contact in the neighbourhood		
Almost daily	24	19.35
Several times a week	21	16.94
Every two or three weeks	21	16.94
Every two or three months	6	4.84
Never	45	36.29
Do not know	1	0.81
Not concerned	6	4.84
**Interview conditions**		
The respondent took part in the interview	117	94.35
Language of the interview		
Luxembourgish	101	81.45
German	10	8.06
French	12	9.68
Other	1	0.81

Notes: full sample of 124 adults with disabilities. *Mean score. **Standard deviation

Table 4 shows QoL average scores by disability ASP profile. The average QoL score of the sample was 3.77 (over a scale from 1 to 5). QoL decreased with an increasing level of intellectual disability. While people with mild intellectual disability, poly-disability or motor disability graded their QoL above the average level, those with severe intellectual disabilities or those who were autistic reported one of the lowest levels of QoL.

**Table 4:** Quality of life of people with disabilities.

Disability – ASP Classification	Freq.	Mean	SD	Min	Max
Others	1	4.07	.	4.07	4.07
Mild intellectual disability	26	3.97	0.51	3.07	4.71
Motor disability	18	3.87	0.6	2.77	4.71
Poly-disability	7	3.79	0.81	2.43	5
Moderate intellectual disability	41	3.75	0.51	2.6	4.57
Severe or profound intellectual disability	26	3.58	0.64	2.21	4.62
Autism	5	3.51	0.73	2.22	4
**Total**	**124**	**3.77**	**0.58**	**2.21**	**5**

### Statistical analyses

Table 5 presents the results of the OLS estimations. Column 1 displays the estimates without accounting for interview conditions and without including interviewer and service provider fixed effects. The final column reports the results from the fully specified model, incorporating all interview controls and fixed effects. There were no significant gender or age differences in terms of QoL. As for civil status, divorced people reported higher levels of QoL than single people (*p* = 0.039). To a lesser extent, being married was also associated with greater QoL scores, compared with being single, but the associated coefficient was only statistically significant at the 10-percent level (*p* = 0.074).

**Table 5:** Factors associated with the quality of life of people with disabilities – OLS estimates.

	(1)			(2)			(3)			(4)		
	β	se	*p*	β	se	*p*	β	se	*p*	β	se	*p*
**Socio-demographics**												
Female	−0.02	0.11	0.864	−0.11	0.11	0.315	−0.02	0.11	0.874	−0.10	0.11	0.355
60+	0.06	0.13	0.640	−0.05	0.13	0.721	0.04	0.13	0.759	−0.07	0.13	0.611
Marital status (ref: single)												
Married	0.59	0.47	0.215	0.96	0.53	0.073	0.58	0.47	0.225	0.98	0.54	0.074
Divorced	0.30	0.20	0.142	**0.42**	**0.20**	**0.035**	0.27	0.20	0.185	**0.43**	**0.20**	**0.039**
Widowed	0.39	0.54	0.463	0.51	0.50	0.306	0.33	0.53	0.537	0.48	0.51	0.343
**Living environment**												
Daycare	**−1.02**	**0.37**	**0.007**	**−0.76**	**0.37**	**0.043**	**−1.01**	**0.38**	**0.009**	**−0.81**	**0.39**	**0.041**
Type of residence (ref: independent housing managed by the service provider)												
In housing within a facility, including only housing owned by a service provider	−0.01	0.23	0.979	0.05	0.24	0.846	−0.03	0.23	0.913	−0.01	0.25	0.961
Private housing with parents	**1.34**	**0.40**	**0.001**	**1.30**	**0.40**	**0.002**	**1.26**	**0.42**	**0.004**	**1.25**	**0.45**	**0.006**
Private housing with family members (but not the parents)	0.76	0.48	0.115	0.21	0.52	0.684	0.61	0.49	0.216	0.11	0.59	0.849
Others	−0.14	0.29	0.636	0.28	0.31	0.365	−0.16	0.29	0.574	0.20	0.32	0.530
Place of residence (ref: residential areas, downtowns, where the rest of the population also lives)												
Outskirts of the city/ suburbs	−0.16	0.12	0.187	−0.01	0.15	0.925	−0.12	0.12	0.343	−0.02	0.16	0.924
Outside of the city	0.08	0.16	0.635	0.22	0.19	0.240	0.10	0.16	0.529	0.22	0.20	0.271
Type of the room (ref: individual with one bed)												
Two persons (separate beds)	0.05	0.43	0.900	−0.06	0.48	0.908	0.12	0.43	0.775	−0.06	0.50	0.901
Two persons (double bed)	**−0.87**	**0.40**	**0.030**	**−1.02**	**0.39**	**0.011**	**−0.92**	**0.39**	**0.022**	**−1.07**	**0.40**	**0.010**
Three or more people (separate beds)	−1.30	0.68	0.057	**−1.73**	**0.70**	**0.016**	**−1.45**	**0.70**	**0.041**	**−1.72**	**0.72**	**0.020**
**Disability**												
Disability classification (ASP Convention) (ref: mild intellectual disability)												
Moderate intellectual disability	−0.11	0.16	0.480	0.08	0.17	0.623	−0.12	0.15	0.436	0.08	0.17	0.632
Severe intellectual disability	−0.09	0.20	0.642	0.30	0.22	0.189	−0.12	0.20	0.551	0.26	0.24	0.284
Poly-disability	0.21	0.29	0.471	0.33	0.31	0.277	0.16	0.29	0.581	0.33	0.32	0.312
Motor disability	0.05	0.25	0.840	0.05	0.35	0.877	0.12	0.26	0.647	0.15	0.37	0.688
Autism	−0.08	0.27	0.785	0.24	0.40	0.550	−0.01	0.28	0.962	0.29	0.42	0.483
Others	−0.51	0.60	0.396	−0.29	0.83	0.725	−0.41	0.60	0.491	−0.11	0.88	0.900
Very limited mobility	0.09	0.16	0.604	0.36	0.18	0.052	0.12	0.18	0.511	0.33	0.20	0.110
Significant medical care needs	0.34	0.32	0.296	0.05	0.35	0.889	0.33	0.32	0.317	0.06	0.35	0.869
Significant needs related to behavioural problems	−0.16	0.13	0.224	−0.23	0.14	0.099	−0.11	0.14	0.429	−0.18	0.15	0.215
Language skills in receptive communication (ref: sufficient)												
Limited	0.09	0.14	0.525	−0.09	0.17	0.587	0.10	0.14	0.496	−0.11	0.18	0.543
None	−0.16	0.27	0.545	−0.46	0.28	0.108	−0.22	0.28	0.427	−0.44	0.31	0.166
Language skills in expressive communication (ref: sufficient)												
Limited	−0.10	0.16	0.519	−0.18	0.16	0.285	−0.15	0.16	0.347	−0.21	0.17	0.212
None	−0.03	0.27	0.904	−0.14	0.27	0.602	−0.01	0.30	0.985	−0.18	0.31	0.563
Objective needs for support	**−0.42**	**0.14**	**0.003**	**−0.50**	**0.14**	**0.001**	**−0.44**	**0.15**	**0.005**	**−0.49**	**0.16**	**0.003**
**Social interactions**												
Working	0.10	0.14	0.469	0.26	0.15	0.082	0.09	0.14	0.520	0.22	0.16	0.157
Frequency of contacts (ref: almost daily)												
Several times a week	−0.22	0.17	0.193	−0.19	0.17	0.256	−0.21	0.17	0.222	−0.19	0.17	0.282
Every two or three weeks	**−0.36**	**0.17**	**0.034**	−0.33	0.17	0.060	**−0.36**	**0.17**	**0.035**	−0.34	0.18	0.060
Every two or three months	**−0.49**	**0.25**	**0.048**	−0.36	0.26	0.161	**−0.50**	**0.24**	**0.045**	−0.40	0.27	0.138
Never	**−0.32**	**0.16**	**0.044**	−0.24	0.15	0.117	−0.29	0.16	0.072	−0.24	0.16	0.154
Do not know	−0.37	0.55	0.505	−0.27	0.53	0.612	−0.33	0.55	0.555	−0.29	0.55	0.597
Not concerned	−0.13	0.28	0.658	−0.15	0.28	0.577	−0.18	0.29	0.535	−0.18	0.30	0.540
Constant	4.96	0.31	0.000	4.93	0.75	0.000	5.12	0.44	0.000	5.23	0.99	0.000
Interview conditions	No			No			Yes			Yes		
Fixed-effects	No			Yes			No			Yes		
Observations	124			124			124			124		
R-squared	0.32			0.43			0.34			0.41		

Notes: full sample of 124 adults with disabilities. Significant threshold of 5%. Fixed effects include surveyor and service providers dummies; interview conditions refer to the language of the interview and the attendance of the respondent.

We aim at capturing a global picture of the disability situation by observing several indicators, including the type of disability, the expression and communication skills as well as mobility-related limitations, significant medical treatment, behavioural troubles and needs for support. While most of these indicators were not statistically significantly correlated with QoL, the needs-for-support variable was negatively associated with QoL (*p* = 0.003): people with higher needs for support reported lower QoL scores.

Several levels of the living environment were considered: the neighbourhood, the accommodation and the bedroom. We also included the accommodation settings, indicating whether the person is in day-care, i.e., spends the day in the premises of the service provider and returns home in the evening, or is in full-time care. There was no significant difference in QoL between living in the outskirt of the city or out of the city, compared to living in a more central area. People living in a private accommodation with parents rated their QoL higher than those living in an accommodation run by the service provider. The estimated coefficient was highly significant (*p* = 0.006) and its magnitude showed a large effect of more than 1 point. People sharing their room with two or more persons (if no separate beds) reported lower levels of QoL. The magnitude of the coefficient increased as more people share the room, showing a detrimental effect on QoL of more than 1.5 points (on a scale from 1 to 5) when the room was shared by three people or more. In addition, people in day-care reported lower QoL (*p* = 0.041) than those in full-time care.

People were asked how often they talk to those in their neighbourhood. There was no difference in QoL when accounting for fixed effects. However, when not including service providers and surveyor dummies, there was a decrease in QoL when interacting with others only every two or three weeks (*p* = 0.035), only every two or three months (*p* = 0.045). When interacting even less, the effect on QoL was still negative but only statistically significant at the 10-percent level (*p* = 0.072). Labour market status was not correlated with QoL.

### Robustness tests

Additional analyses were conducted to assess the robustness of our findings using alternative subsamples and sensitivity checks. First, given the high proportion of individuals with intellectual disabilities in our sample, including autistic individuals, we performed a focused analysis on this subgroup. Second, in some cases, the respondent did not personally participate in the interview and was replaced by a proxy, a trusted person who responded on their behalf. This may lead to over- or under-reporting bias; thus, we repeated the analysis using only the subsample of self-respondents. Finally, we examined the sensitivity of our results to the response rate threshold imposed on the QoL scale. Specifically, we re-ran the analysis on two restricted subsamples: those who answered at least 50% and at least 70% of the QoL-related items. The results of these analyses are presented in S4 Appendix, Table S4A and confirm the reliability of the main findings.

## Discussion

In this study, we show that support needs, living environment and social contact frequency significantly influence the QoL of people with disabilities. People with higher support needs report lower QoL scores, as do people who share a room with other people and people who have infrequent contact with their neighbourhood. To our knowledge, this is the first study to examine the factors associated with the QoL of people with disabilities in Luxembourg, contributing to filling an important gap in the literature on this topic in small, high-income countries. The Luxembourgish case is nevertheless relevant to broader European debates on disability policy, as the country combines strong welfare provision and formal commitment to the UNCRPD with a continued reliance on provider-led residential and occupational support systems.

Most studies on QoL of people with disabilities focus on a specific disabled population, and therefore stop short of examining any potential differences in QoL between disability profiles [10,24]. Our findings indicate that when different disability profiles are considered, most of the determinants related to the disability situation turn out not to be statistically significantly correlated with QoL. In particular, neither the disability profile (e.g., physical disability, intellectual disability) nor the expressive or receptive communication skills seem to affect the level of QoL, suggesting that the disability itself does not correlate with the QoL of people with disabilities. This may be interpreted as in line with the spirit of the social model of disability supported by the UNCRPD, going beyond the traditionally narrow medical perspective. This finding should not be interpreted as evidence that these characteristics are unrelated to QoL. Rather, in the present sample, and conditional on the other variables included in the model, the data did not provide sufficient statistical evidence of such associations. Given the modest sample size and small numbers in some disability subgroups, the possibility of meaningful associations cannot be ruled out.

By contrast, support needs, which represent the extent to which people with disabilities perceive themselves to be able to carry out daily tasks with the support they need, are highly correlated with QoL. This may provide a more accurate picture where people with disabilities are part of an environment that may (or may not) be designed to meet their needs, as described in the social-ecological perspective of disability [32]. The findings align with socio-ecological approaches to disability suggesting that QoL is shaped less by impairment characteristics themselves than by environmental conditions, autonomy, quality of support, and opportunities for participation in community life [26,33]. According to this view, disability is less linked to medical condition but rather to a state of functioning characterised by a persistent and significant difference between a person’s competences and the demands of the environment and participation in an inclusive society, in which they can live [34–36]. In this context, support paradigm becomes particularly relevant, as it emphasises identifying the resources a person needs to live a meaningful and productive life in an inclusive environment [37]. From this link between the support and socio-ecological paradigms, another explanation for the non-significant relationship between most disability-related determinants and QoL can be found in the way people with disabilities are supported in their accommodation settings. People with disabilities in our study sample live part-time or full-time in community-based residential setting and are supported 24-hours by staff. It may be plausible to assume that these staff place more emphasis on supporting people with disabilities in their daily tasks independently of their disability-related condition. This may influence the way people with disabilities perceive their situation as requiring a certain level of personalised support.

Previous studies have analysed the role of the living environment in affecting QoL. For instance, it was found that people with disabilities living in larger and well-furnished spaces report greater levels of satisfaction and QoL [21]. Our results show that living in a private home with parents is associated with an increase in QoL, compared to living in an accommodation run by the service provider. Private living arrangements may not only enhance the sense of freedom and autonomy but may also promote the perception of inclusion. In this context, studies have shown that participation in the community can positively impact on QoL [38,39]. As for the room characteristics, which is the smallest – perhaps most personal space in a house –, people with disabilities report lower QoL as more people share the room. This could depict a situation where co-residents lack privacy or where the risk of conflict is increased [21]. It is also in line with previous studies [25,40] showing that people with intellectual disabilities may have their rights better respected in a residential living environment with fewer residents. Previous European research has highlighted that institutional culture may persist even in community-based settings when opportunities for privacy, personal choice, and self-determination remain limited [33,41].

We also found that people with disabilities in day-care report lower QoL scores than those in full-time institutional living. Most people in day-care travel to and from their homes at least several times a week. This changing environment can create a feeling of insecurity that may unsettle them. In addition, people with disability in day-care may tend to live in a more flexible and personalised environment when they are at home. They might also mix relatively more with people without disability. The lower QoL of day-care residents may come from their awareness of an alternative way of living. Another explanation is that people with disabilities in day-care have a higher degree of disability and lower financial resources on average. In addition, the daily activities and support offered in the day-care setting tend to be less personalised. In day-care, people with disabilities are often supported in a group setting and they follow group activities. Due to limited resources, they often do not have the opportunity to pursue an individualised activity program. These findings are consistent with European research showing that community-based and individualised living arrangements are generally associated with higher QoL, particularly when they promote autonomy, privacy, and participation in community life [26,42]. While the setting and the type of accommodation are correlated with QoL, the dwelling location has no effect on QoL, as also shown by [24]. Specifically, there is no difference in QoL for people with disabilities living in the city centre, in the suburbs or in a rural area. This could be explained by a low level of participation in community life, outside the institution or the home.

QoL also relates to social interactions, in that those with less frequent contact with the neighbourhood report lower QoL, specifically for people with intellectual disabilities. This is consistent with the social model of disability. Other studies have also looked at employment as a measure of social interaction, exploring to what extent it might contribute to QoL [5,21–23]. Perhaps, surprisingly, in contrast to the results of some other studies [5,21–23], the present results show that employment (working vs non-working) does not appear to make a difference to QoL. Research shows that the mere fact of being in work is not automatically associated with a sense of social participation [43]. Rather, what does appear to create the sense of participation is the context of daily activities and social interactions at work – a result that might be partially attributable to the Luxembourgish context. Hence, to be aligned with the UNCRPD and with the aim of creating a more inclusive labour market, Luxembourg has set up workplaces adapted to people with disabilities. These workshops promoting professional inclusion were intended to be a lever to enter the main labour market and hence had initially been conceived as temporary [44]. However, people with disabilities are increasingly oriented towards these protected workplaces and risk staying there permanently, and without options for promotion. In our sample, all of the employed individuals work in these workshops allegedly seeking to promote inclusion, even if ultimately, they appear to lead to workplaces that promote social segregation. That said, there is a general challenge in unambiguously defining and measuring social inclusion, as much of the previous research has focused on objective measures that do not analyse what participation means from the subjective perspective of a people [45,46]. This may also explain why employment status alone was not associated with QoL in the present study**.** Recent research further emphasises that improvements in QoL depend not only on physical integration into community settings or employment structures, but also on opportunities for decision-making, meaningful participation, autonomy, and social relationships [47].

In interpreting our results, it is important to also acknowledge the study limitations. The legal framework in Luxembourg does not (yet) allow for a national registry of people with disabilities. As a result, it was only possible to contact people with disabilities cared for under the Convention ASP through the service providers hosting them. This has several implications: first, focusing on the ASP users, our results are not necessarily representative of the overall disabled population of Luxembourg, with relatively few representations of participants with autism or multiple disabilities. It would be interesting to reproduce such a study with a representative sample for Luxembourg. Second, it introduces an inherent selection bias in that only those who live in – or attend – support systems of a service provider– part- or full-time – are observed. Hence, people with disabilities living at home in relative autonomy, and with perhaps less severe disabilities, are not included in our sample. As a result, people in our sample may have more difficulties in communicating or expressing themselves and therefore require the presence of a proxy, a trusted person who responded on their behalf. Moreover, interviews were administered by trained interviewers who were instructed to monitor response consistency and identify potential inconsistencies across related items. Although these procedures helped support the reliability and validity of the data collected, differences in participants’ understanding of certain questionnaire items cannot be entirely excluded. Finally, a notable limitation of our study lies in the relatively low internal consistency of the QoL scale used, with a Cronbach’s alpha of 0.57. Although our QoL measure was grounded in a well-established theoretical framework [29], the scale showed only modest reliability. This indicates that the selected items may not fully capture a cohesive underlying construct and could benefit from further refinement. Future research would benefit from using a more comprehensive validated QoL instrument to strengthen the analysis and provide a more robust understanding of the multiple factors influencing the QoL of persons with disabilities.

## Conclusion

Our findings lend support to the view that to improve the QoL of people with disabilities, authorities and policymakers should recognise the importance of neighbourhood interactions on QoL and propose community-level interventions and social engagement programmes to promote greater social interaction within the community and neighbourhood. These interventions should not only target people with disabilities but also the wider community by developing and supporting community engagement programs that increase the frequency of interactions between people with disabilities and without disabilities. On an individual level, interventions to enhance social and expressive skills could also be considered to improve the interaction of people with disabilities with more people in the community. Moreover, individualised support, considering individual support needs such as personal assistance, could help people with disabilities in improving their QoL.

## Supporting information

S1 Appendix The ASP convention. (DOCX)

S2 Appendix Final version of the questionnaire. (DOCX)

S3 Appendix ASP and ICD classification. (DOCX)

S4 Appendix Robustness tests. (DOCX)
