# Supplementary material for: Factors associated with the quality of life of people with disabilities living in institutions or attending day care in Luxembourg
Source: PLoS One. 2026 Sep 8;21(9):e0356610. doi: 10.1371/journal.pone.0356610 (PMC13552743; doi:10.1371/journal.pone.0356610)
Supplement: Supplementary file 1 [file pone.0356610.s001.docx]

# S1 Appendix. The ASP Convention

The ASP Convention is an agreement, which defines the socio-pedagogical support for people with disabilities specific to Luxembourg. It aims to offer people with disabilities a living context that contributes to their well-being, autonomy and inclusion. Furthermore, the 2018 ASP Agreement regulates the relationship between this ministry, which is responsible for many aspects of the lives of people with disabilities, and service providers who work in the disability sector and provide support services to people with disabilities who are either in day care or in full-time institutions. The Luxembourg Convention describes that people with disabilities, minors and adults who need socio-educational support related to their disability are part of this convention. The ASP -convention is based on the framework law of 8 September 1998 known as the "ASFT Law "9 (social, family and therapeutic activity) and amended in 2011, as well as on the Grand-Ducal regulation of 9 January 2001 relating to services agreed by the Ministry for the Family, Integration and the Greater Region.
