# Supplementary material for: Factors associated with the quality of life of people with disabilities living in institutions or attending day care in Luxembourg
Source: PLoS One. 2026 Sep 8;21(9):e0356610. doi: 10.1371/journal.pone.0356610 (PMC13552743; doi:10.1371/journal.pone.0356610)
Supplement: Supplementary file 2 [file pone.0356610.s002.docx]

# S2 Appendix. Final version of the questionnaire

**Socio-education program beneficiaries' point of view (survey of people with disabilities)**

user code: ______________________________

service provider: _________________________

interviewer: _____________________________

Introduction section

| A. **Place of residence** (interviewer completes details herself) | | **(one answer only)** | |
| --- | --- | --- | --- |
| a) the place of residence is located in an area where the rest of the population also lives, e.g. a residential area, in the city centre or in the heart of the village | |  | |
| b) the place of residence is on the outskirts of a town (suburb)/village | |  | |
| c) the place of residence is outside a town/village | |  | |
| B. **The disabled person occupies a room** | | **(one answer only)** | |
| a) of one person | |  | |
| b) for two people (two separate beds) | |  | |
| c) for two people (one bed) | |  | |
| d) three or more people (each in their own bed) | |  | |
|  | C. **Activity of the disabled person CHILD/ADOLESCENT (if year of birth>=2003)** | **yes** | **no** |
|  | a) the disabled child/adolescent attends a class in an inclusive school |  |  |
|  | b) the disabled child/adolescent attends a class in a skills centre |  |  |
|  | c) the disabled child/adolescent receives vocational training in an inclusive school centre |  |  |
|  | d) the disabled child/adolescent undergoes vocational training in a vocational training centre |  |  |
|  | e) the disabled child/adolescent goes to a day care services |  |  |
|  | f) other (please specify) |  |  |

| D. **Information about the disabled person (respondent)** | |
| --- | --- |
| a) weight (in kg) |  |
| b) height (in cm) |  |

Note who gave this information: _____________

| E. **Use of technology and communication (abilities of people with disabilities)** | **yes** | **no** | **With internet access** |
| --- | --- | --- | --- |
| a) The person uses a phone |  |  |  |
| b) The person uses a smartphone |  |  |  |
| c) The person uses computers |  |  |  |

| F. **Marital status (if year of birth<2003)** | **(one answer only)** |
| --- | --- |
| a) single (E.g.: I am alone; I have a boyfriend/girlfriend) |  |
| b) civil partnership |  |
| c) married |  |
| d) divorced |  |
| e) widow |  |
| f) do not know |  |

Main questions

1. Social participation (the people you meet)

Normally, before COVID restrictions:

1.1. How often do you talk to people in your neighbourhood? (Neighbours: people living in the same area, in houses/apartments close to where you live)

b) almost every day
c) several times a week
d) every two or three weeks
e) every two or three months
f) never
g) do not know
h) do not want to answer
i) not concerned
   1.2. How often do you see friends or relatives who do not live with you?
b) almost every day
c) several times a week
d) every two or three weeks
e) every two or three months
f) never
g) do not know
h) do not want to answer
i) not concerned
   1.3. Do you have any other activities outside the facility? E.g.: voluntary work, craft classes, village club, leisure activity, shopping, neighbourhood meal, sports club, religious community, etc.
b) almost every day
c) several times a week
d) every two or three weeks
e) every two or three months
f) never (go to question 2.1)
g) do not know (go to question 2.1)
h) do not want to answer (go to question 2.1)
i) not concerned (go to question 2.1)
   1.3.1. Who will accompany you on these activities?
b) parents
c) other family members
d) friends
d) paid caregivers
e) other .......
f) no one
2. Skills (what I have learnt, what I know how to do)
   2.1. In which areas have you made progress or learnt new things in the last two years? Have you learnt anything new in the last two years? (at work, at the day care services, leisure time, at home, in the garden)

a) Themes or areas______________________________________________________

b) do not know

c) do not want to answer

d) not concerned

2.2. Is there anything you are particularly good at?

a) yes (which one? ...........)

b) no

c) do not know

d) do not want to answer

e) not concerned

2.3. How often do you watch television or Internet videos?

a) almost every day
b) several times a week
c) every two or three weeks
d) every two or three months
e) never
f) do not know
g) do not want to answer
h) not concerned
   2.4. How often do you read or look at a book, comic strip, magazine or newspaper?
a) almost every day
b) several times a week
c) every two or three weeks
d) every two or three months
e) never
f) do not know
g) do not want to answer
h) not concerned
   2.5. How often do you listen to the news (the Journal, the Heielei, the 5 minutes)?
a) almost every day
b) several times a week
c) every two or three weeks
d) every two or three months
e) never
f) do not know
g) do not want to answer
h) not concerned

3. Health questions
   3.1. Are you involved in any sporting or outdoor activities? Such as football, swimming, cycling, hiking, trampoline, skiing, etc.
b) almost every day
c) several times a week
d) every two or three weeks
e) every two or three months
f) never
g) do not know
h) do not want to answer
i) not concerned
   3.2. How far can you walk (or wheelchair), on a flat road, without taking a break and without feeling sore?

(Here you can also ask the time: ¼ hour are more than 500m)

a) no
b) less than 100 metres
c) less than 500 metres
d) more than 500 metres
e) do not know
f) do not want to answer
g) not concerned
   3.3. In the last 12 months, have you been to hospital/clinic for one night or more?
b) yes
c) no
d) do not know
e) do not want to answer
f) not concerned
   3.4. Do you take medication regularly?
b) yes
c) no
d) do not know
e) do not want to answer
f) not concerned
4. Material situation (for children and teenagers, suggest answers in **bold**)
   4.1. Do you know your monthly (weekly) **income/pocket** money?
b) yes
c) no
d) do not know
e) do not want to answer
f) not concerned
   4.2. Can you buy everything you want/need with this money?
b) yes
c) no
d) do not know
e) do not want to answer
f) not concerned
   4.3. Is the money you have enough to live on generally?
a) yes
b) no
c) do not know
d) do not want to answer
e) not concerned

5. Autonomy

5.1. I can do a lot of housework myself.

a) yes	d) do not want to answer
b) no	e) not concerned
c) do not know

5.2. For certain activities I need guidance and support (e.g. leisure activities, cultural or religious activities, sport, visiting friends).

a) yes	d) do not want to answer
b) no	e) not concerned
c) do not know
   5.3. I can use public transport myself (e.g. bus, train, tram).
a) yes	d) do not want to answer
b) no	e) not concerned
c) do not know

5.4. In my free time (leisure), I can do the things that are important to me most of the time (my favourite activities).

a) yes	d) do not want to answer
b) no	e) not concerned
c) don't know
6. Support needs

Interviewer provides instruction on how to answer the questions in this section using an example. For children and teenagers, suggest questions in **bold**. Rewording suggestions are in *italic*.

| We all need help when things are too difficult for us. Now I would like to know how much help you generally need to do different things. I am going to give you a situation, and each time you tell me whether you can do these things on your own, whether you need a little help or a lot of help, or whether someone has to do it for you. | I can do it alone | Sometimes I need help | I always need help | Someone has to do it for me | I do not know | I do not want to answer | Not concerned |
| --- | --- | --- | --- | --- | --- | --- | --- |
| e) Getting dressed in the morning |  |  |  |  |  |  |  |
| f) Taking a shower or a bath |  |  |  |  |  |  |  |
| g) Going to the toilet |  |  |  |  |  |  |  |
| h) Ordering something to eat or drink in a bar/restaurant |  |  |  |  |  |  |  |
| i) Doing the laundry / **Looking after my things** |  |  |  |  |  |  | **E/A** |
| j) Preparing a sandwich |  |  |  |  |  |  |  |
| k) Eating on my own |  |  |  |  |  |  |  |
| l) Knowing what is on TV |  |  |  |  |  |  |  |
| m) Make an appointment (e.g. with a doctor) / **Arrange a meeting with my friends** |  |  |  |  |  |  | **E/A** |
| n) Going shopping at the supermarket / **buying sweets or toys** |  |  |  |  |  |  |  |
| o) Having good relationships with other people / *making friends* |  |  |  |  |  |  | **E/A** |
| p) Communicating personal wishes and desires |  |  |  |  |  |  |  |

7. Quality of life

Interviewer provides instruction on how to answer the questions in this section using an example. For children and teenagers, suggest answers in **bold**. Rewording suggestions are in *italic*.

|  | 2. 3. 4. 5. 6. 7.   7.1.QoL: Well-being (**P**erson/**E**nvironment) | Absolutely true | Quite true | Neither | Quite wrong | Completely wrong | I do not know | I do not want to answer | Not concerned |
| --- | --- | --- | --- | --- | --- | --- | --- | --- | --- |
| BE-2 | My life is very stressful |  |  |  |  |  |  |  |  |
| BP+6 | Most of the things that happen to me are nice and make me happy |  |  |  |  |  |  |  |  |
| BP+8 | I am happy with my state of health / I am in good health |  |  |  |  |  |  |  |  |
| BP-9 | There are often disputes and conflicts *(noise)* where I live |  |  |  |  |  |  |  |  |
|  | 1. 2. 3. 4. 5. 6. 7. 7.1.    7.2. QoV: **I**nteraction/**C**ompetence |  |  |  |  |  |  |  |  |
| I+14 | I get on well with the people who look after me *(the staff)* |  |  |  |  |  |  |  |  |
| I-15 | I am considered more of a stranger *(in my group I tend to be excluded)* |  |  |  |  |  |  |  |  |
| C+17 | I help a lot with household chores |  |  |  |  |  |  |  |  |
| C-19 | There are many things that I cannot do as well as others (negation, possibly change) *I can do many things as well as others.* |  |  |  |  |  |  |  |  |
|  | 7.3. QoL: **A**utonomy**/S**elf-sufficiency |  |  |  |  |  |  |  |  |
| S+21. | Most of the time, I can use my (**pocket**) money as I wish |  |  |  |  |  |  |  |  |
| S+23. | I decide for myself what I want to do (e.g. what time I go to bed, what I want to eat, when my friends come to visit). |  |  |  |  |  |  |  |  |
| A-28. | My educators (**parents**) (*staff*) often decide what I should do |  |  |  |  |  |  |  |  |
| A-30. | What is important to me in my life is often not respected/supported (negation, possibly change) *Staff are not interested in what is important to me.* |  |  |  |  |  |  |  |  |
|  | 7.4. QoL: **S**ocial Inclusion/**C**ommunity **P**articipation |  |  |  |  |  |  |  |  |
| PC+31. | I like being part of a club (sport, music, church) and I am an active member. |  |  |  |  |  |  |  |  |
| AS+34. | Being able to talk to my neighbours is very important to me |  |  |  |  |  |  |  |  |
| PC-36. | I feel rejected by many people in our village (town, neighbourhood) |  |  |  |  |  |  |  |  |
| AS-40. | The way people treat me is not right, not just *not OK, not* right |  |  |  |  |  |  |  |  |

8. Self-determination

Interviewer provides instruction on how to answer the questions in this section using an example. For children and teenagers, suggest answers in **bold**. Rewording suggestions are in *italic*.

|  | 8.1. Perceived **c**ompetence | Absolutely true | Quite true | Neither | Quite wrong | Completely wrong | I don't know | I don't want to answer | Not concerned |
| --- | --- | --- | --- | --- | --- | --- | --- | --- | --- |
| AD-C+1 | I am often told that I do things well (the interviewer asks for examples*), I often receive compliments* |  |  |  |  |  |  |  |  |
| AD -C-2 | I hardly ever learn new things |  |  |  |  |  |  |  |  |
| AD -C+3 | I often have the opportunity to show that I can do things |  |  |  |  |  |  |  |  |
| AD -C-4 | Most of the time, I feel like I have done nothing (accomplished)  *I often feel like I have achieved nothing* |  |  |  |  |  |  |  |  |
|  | 8.2. Perceived **a**utonomy |  |  |  |  |  |  |  |  |
| AD -A+1 | I can decide for myself how I want to live |  |  |  |  |  |  |  |  |
| AD -A-2 | My ideas and opinions are often not taken seriously, *I am often not taken seriously* |  |  |  |  |  |  |  |  |
| AD -A-3 | I often have to do what I am told in my daily life |  |  |  |  |  |  |  |  |
| AD -A+4 | I can be myself (be who I want to be) |  |  |  |  |  |  |  |  |
|  | 8.3 Perceived **s**ocial **i**nclusion |  |  |  |  |  |  |  |  |
| AD -IS+1 | I am happy with the people around me |  |  |  |  |  |  |  |  |
| AD -IS-2 | A lot of people are not really interested in me |  |  |  |  |  |  |  |  |
| AD -IS+3 | Most of the time, people *(staff)* pay attention to how I am doing |  |  |  |  |  |  |  |  |
| AD -IS-4 | People are often unkind to me, *people outside are nice to me* |  |  |  |  |  |  |  |  |

9. Temperament and satisfaction

Interviewer provides instruction on how to answer the questions in this section using an example. For children and teenagers, suggest answers in **bold**. Rewording suggestions are in *italic*.

|  | 9.1. Subjective **v**itality | Absolutely true | Quite true | Neither | Quite wrong | Completely wrong | I don't know | I don't want to answer | Not concerned |
| --- | --- | --- | --- | --- | --- | --- | --- | --- | --- |
| VI+1. | I always look forward to the next day |  |  |  |  |  |  |  |  |
| VI-2. | I often do not feel like doing anything |  |  |  |  |  |  |  |  |
| VI+3 | Sometimes I have so much energy that I do not know what to do with it (e.g. I want to run around, jump...), *sometimes I am so fit that I need to let off steam.* |  |  |  |  |  |  |  |  |
| VI-4 | I prefer to be alone |  |  |  |  |  |  |  |  |
| VI-5. | No matter what I say, it is useless, no one listens to me |  |  |  |  |  |  |  |  |
| VI+6. | I have lots of projects in my life, lots of things to do. *In my life I still have a lot planned, I still want to do a lot.* |  |  |  |  |  |  |  |  |
|  | 9.2. Satisfaction |  |  |  |  |  |  |  |  |
| S+1. | I am satisfied with where I live, with my home... |  |  |  |  |  |  |  |  |
| S+2. | I am satisfied with the leisure activities I can enjoy... |  |  |  |  |  |  |  |  |
| S+3. | I am satisfied with my job**/hosting** structure.... *Day Care services* |  |  |  |  |  |  |  |  |
| S-4. | A few years ago, I was much more satisfied with my life... *I used to be happier / when I was working, I was happier* |  |  |  |  |  |  |  |  |
| S+5. | I am treated *fairly* by my teachers... *(staff)* |  |  |  |  |  |  |  |  |
| S+6. | Overall, I am satisfied with my life |  |  |  |  |  |  |  |  |

10. Satisfied needs and unmet and unsatisfied needs (what I lack and what I will need)

Interviewer provides instruction on how to answer the questions in this section using an example. For children and teenagers, suggest answers in **bold**. Rewording suggestions are in *italic*.

|  |  | Absolutely true | Quite true | Neither | Quite wrong | Completely wrong | I don't know | I don't want to answer | Not concerned |
| --- | --- | --- | --- | --- | --- | --- | --- | --- | --- |
| BNC5+41 | My future projects are well supported |  |  |  |  |  |  |  |  |
| BI1-42 | I would like to have a healthier *diet, I would like to eat better,* healthier *food* |  |  |  |  |  |  |  |  |
| BI2-43 | I do not feel safe at home (negation, change if necessary) |  |  |  |  |  |  |  |  |
| BNC2-44 | I would like to have more freedom with my (**pocket**) money, like other people (non-disabled people). |  |  |  |  |  |  |  |  |
| BNC4+45 | I can do things that are important to me, *I can do things that are important to me* |  |  |  |  |  |  |  |  |
| BI3+46 | I would like to spend more time with people who are not disabled |  |  |  |  |  |  |  |  |
| BNC1+47 | I would like to be left alone more often |  |  |  |  |  |  |  |  |
| BI4-48 | Educators are often not interested in what I want (or what I say, or what is important to me) *Staff is not interested in me and what I want to do* |  |  |  |  |  |  |  |  |
| BF5+49 | I would like to be more independent / I am **looking forward to becoming more independent** |  |  |  |  |  |  |  |  |
| BF4-50 | I am often offered activities that do not really interest me |  |  |  |  |  |  |  |  |
| BF5-51 | I can hardly change anything in my life |  |  |  |  |  |  |  |  |
| BF3-52 | I am short of good friends |  |  |  |  |  |  |  |  |
| NM4-53 | I would like my *parents/caregivers* to understand me better |  |  |  |  |  |  |  |  |

Finally, two open questions :

10.1. What I miss most, what I really need and what I would most like to have:

_____________________________________________________________________________________________________________________________________________________________________________________________________________________________________________________________________________________________________________________________________________________________________________________________

10.2. What I do not need at all, what annoys me and what I do not need at all:

_____________________________________________________________________________________________________________________________________________________________________________________________________________________________________________________________________________________________________________________________________________________________________________________________

General comments on the interview:

____________________________________________________________________________________________________________________________________________________________________________________________________________________________________________________________________________________________________________________________________________
