# Supplementary material for: Factors associated with the quality of life of people with disabilities living in institutions or attending day care in Luxembourg
Source: PLoS One. 2026 Sep 8;21(9):e0356610. doi: 10.1371/journal.pone.0356610 (PMC13552743; doi:10.1371/journal.pone.0356610)
Supplement: Supplementary file 3 [file pone.0356610.s003.docx]

# S3 Appendix. ASP and ICD classifications

Table S3A. Corresponding categories between the ASP and the ICD classifications

| **ASP** | **ICD-11** |
| --- | --- |
| Mild intellectual | Disorders of intellectual development, mild (6A00.0) |
| Medium intellectual | Disorders of intellectual development, moderate (6A00.1) |
| Severe intellectual | Disorders of intellectual development, severe and profound (6A00.2 & 6A00.3) |
| Explanation (not ICD-11) | |
| Poly-disabilities | Multiple disabilities – disabilities typically affecting the motor system with in addition sensory and/or intellectual disabilities |
| Motor | Disabilities related to the motor system with intellectual functioning in the normal range |
| Autism | Autism spectrum disorders with intellectual disabilities and frequent behaviour problems |
| Other | Attention-deficit/hyperactivity disorder (ADHD), young children without diagnosis |
