# Supplementary material for: Factors associated with the quality of life of people with disabilities living in institutions or attending day care in Luxembourg
Source: PLoS One. 2026 Sep 8;21(9):e0356610. doi: 10.1371/journal.pone.0356610 (PMC13552743; doi:10.1371/journal.pone.0356610)
Supplement: Supplementary file 4 [file pone.0356610.s004.docx]

# S4 Appendix. Robustness tests

Table S4A. Determinants of quality of life over alternative subsamples of people with disabilities – OLS estimates.

|  | People with intellectual disability | | | Without proxy | | | 70% response rate | | | 50% response rate | | |
| --- | --- | --- | --- | --- | --- | --- | --- | --- | --- | --- | --- | --- |
|  | β | se | *p* | β | se | *p* | β | se | *p* | β | se | *p* |
| **Socio-demographics** |  |  |  |  |  |  |  |  |  |  |  |  |
| Female | -0.18 | 0.12 | 0.138 | -0.12 | 0.11 | 0.265 | -0.05 | 0.11 | 0.635 | -0.12 | 0.11 | 0.254 |
| 60+ | -0.11 | 0.15 | 0.446 | -0.08 | 0.13 | 0.535 | -0.08 | 0.14 | 0.569 | -0.10 | 0.13 | 0.428 |
| Marital status (ref: single) |  |  |  |  |  |  |  |  |  |  |  |  |
| Married | -0.65 | 1.13 | 0.568 | **1.40** | **0.57** | **0.017** | 1.04 | 0.54 | 0.060 | 0.91 | 0.53 | 0.091 |
| Divorced | 0.34 | 0.22 | 0.124 | **0.43** | **0.20** | **0.031** | 0.41 | 0.21 | 0.054 | **0.45** | **0.20** | **0.028** |
| Widowed | 0.35 | 0.50 | 0.482 | 0.47 | 0.49 | 0.336 | 0.48 | 0.51 | 0.345 | 0.51 | 0.51 | 0.320 |
| **Living environment** |  |  |  |  |  |  |  |  |  |  |  |  |
| Daycare | -0.12 | 0.73 | 0.869 | **-0.85** | **0.37** | **0.026** | -0.72 | 0.39 | 0.074 | -0.86 | 0.38 | **0.028** |
| Type of residence (ref: Independent  housing managed by the service provider) |  |  |  |  |  |  |  |  |  |  |  |  |
| in housing within a facility, including only  owned by a service provider | -0.02 | 0.27 | 0.939 | -0.03 | 0.24 | 0.916 | 0.02 | 0.25 | 0.945 | -0.07 | 0.24 | 0.781 |
| Private housing parents | 0.73 | 0.61 | 0.241 | **1.24** | **0.44** | **0.006** | **1.22** | **0.45** | **0.009** | **1.18** | **0.44** | **0.009** |
| Private housing with family members  (but not the parents) |  |  |  | -0.40 | 0.65 | 0.546 | 0.07 | 0.60 | 0.901 | -0.13 | 0.55 | 0.814 |
| Others | 0.10 | 0.34 | 0.773 | 0.20 | 0.31 | 0.525 | 0.35 | 0.36 | 0.335 | 0.13 | 0.32 | 0.693 |
| Place of residence (ref: residential areas,  downtowns, where the rest of the population also lives) |  |  |  |  |  |  |  |  |  |  |  |  |
| Outskirts of the city/ suburbs | 0.14 | 0.18 | 0.429 | 0.08 | 0.15 | 0.601 | -0.01 | 0.16 | 0.961 | -0.04 | 0.16 | 0.812 |
| Outside of the city | 0.31 | 0.26 | 0.238 | 0.18 | 0.21 | 0.395 | 0.27 | 0.20 | 0.181 | 0.19 | 0.19 | 0.304 |
| Type of the room (ref : individual with one bed) |  |  |  |  |  |  |  |  |  |  |  |  |
| Two persons (separate beds) | 0.22 | 0.68 | 0.742 | -0.12 | 0.48 | 0.810 | -0.10 | 0.50 | 0.835 | 0.02 | 0.49 | 0.964 |
| Two persons (double bed) |  |  |  | **-1.31** | **0.39** | **0.001** | **-1.09** | **0.40** | **0.009** | **-0.97** | **0.39** | **0.015** |
| Three or more people (separate beds) |  |  |  | **-2.04** | **0.72** | **0.006** | **-1.80** | **0.72** | **0.016** | **-1.60** | **0.71** | **0.026** |
| **Disability** |  |  |  |  |  |  |  |  |  |  |  |  |
| Disability classification (ASP Convention) (ref: mild intellectual) |  |  |  |  |  |  |  |  |  |  |  |  |
| Moderate intellectual disability |  |  |  | 0.08 | 0.17 | 0.615 | 0.07 | 0.18 | 0.699 | 0.07 | 0.17 | 0.689 |
| Severe intellectual disability |  |  |  | 0.24 | 0.24 | 0.325 | 0.31 | 0.25 | 0.215 | 0.17 | 0.23 | 0.462 |
| Poly-disability |  |  |  | 0.50 | 0.32 | 0.119 | 0.28 | 0.33 | 0.400 | 0.34 | 0.31 | 0.274 |
| Motor disability |  |  |  | 0.28 | 0.37 | 0.460 | 0.06 | 0.38 | 0.867 | 0.16 | 0.37 | 0.669 |
| Others |  |  |  | 0.41 | 0.90 | 0.652 | -0.34 | 0.92 | 0.711 | 0.51 | 0.54 | 0.343 |
| Autism |  |  |  | -0.18 | 0.45 | 0.687 | 0.06 | 0.70 | 0.928 | 0.19 | 0.41 | 0.637 |
| Very limited mobility | 0.34 | 0.22 | 0.134 | 0.22 | 0.20 | 0.260 | 0.33 | 0.21 | 0.129 | 0.29 | 0.20 | 0.151 |
| Significant medical care needs | 0.04 | 0.42 | 0.931 | -0.02 | 0.34 | 0.955 | -0.04 | 0.36 | 0.907 | 0.01 | 0.35 | 0.988 |
| Significant needs related to behavioural problems | -0.19 | 0.16 | 0.233 | -0.04 | 0.16 | 0.814 | -0.12 | 0.16 | 0.461 | -0.19 | 0.15 | 0.189 |
| Language skills in receptive communication skills (ref: sufficient) |  |  |  |  |  |  |  |  |  |  |  |  |
| Limited | -0.03 | 0.18 | 0.847 | -0.12 | 0.18 | 0.508 | -0.07 | 0.19 | 0.690 | -0.12 | 0.18 | 0.513 |
| None | -0.23 | 0.32 | 0.481 | -0.39 | 0.31 | 0.204 | -0.20 | 0.36 | 0.585 | -0.28 | 0.28 | 0.323 |
| Language skills in expressive communication skills (ref: sufficient) |  |  |  |  |  |  |  |  |  |  |  |  |
| Limited | -0.28 | 0.18 | 0.127 | -0.32 | 0.17 | 0.063 | -0.23 | 0.17 | 0.180 | -0.24 | 0.17 | 0.165 |
| None | -0.27 | 0.36 | 0.458 | -0.31 | 0.31 | 0.330 | -0.38 | 0.38 | 0.321 | -0.23 | 0.31 | 0.451 |
| Objective needs for support | **-0.64** | **0.22** | **0.004** | **-0.42** | **0.15** | **0.009** | **-0.45** | **0.16** | **0.008** | **-0.49** | **0.16** | **0.003** |
| **Social interactions** |  |  |  |  |  |  |  |  |  |  |  |  |
| Working | 0.01 | 0.19 | 0.945 | 0.21 | 0.15 | 0.173 | 0.28 | 0.17 | 0.099 | 0.17 | 0.15 | 0.272 |
| Frequency of contacts (ref: almost daily) |  |  |  |  |  |  |  |  |  |  |  |  |
| Several times a week | -0.12 | 0.18 | 0.501 | -0.18 | 0.16 | 0.279 | -0.16 | 0.18 | 0.379 | -0.23 | 0.17 | 0.169 |
| Every two or three weeks | **-0.44** | **0.20** | **0.030** | -0.34 | 0.17 | 0.054 | -0.29 | 0.18 | 0.119 | **-0.38** | **0.17** | **0.033** |
| Every two or three months | **-0.74** | **0.30** | **0.017** | -0.40 | 0.25 | 0.117 | -0.33 | 0.27 | 0.225 | -0.46 | 0.26 | 0.083 |
| Never | -0.30 | 0.16 | 0.071 | -0.22 | 0.16 | 0.168 | -0.15 | 0.17 | 0.378 | -0.28 | 0.16 | 0.080 |
| Do not know | -0.37 | 0.54 | 0.498 | -0.39 | 0.53 | 0.467 |  |  |  | -0.33 | 0.53 | 0.534 |
| Not concerned | 0.06 | 0.34 | 0.851 | -0.04 | 0.31 | 0.906 | -0.05 | 0.32 | 0.869 | -0.16 | 0.29 | 0.584 |
| Constant | -0.12 | 0.18 | 0.501 | 5.60 | 0.87 | 0.000 | 4.70 | 1.05 | 0.000 | 6.03 | 0.73 | 0.000 |
| Interview conditions | Yes |  |  | Yes |  |  | Yes |  |  | Yes |  |  |
| Fixed-effects | Yes |  |  | Yes |  |  | Yes |  |  | Yes |  |  |
| Observations | 98 |  |  | 117 |  |  | 115 |  |  | 127 |  |  |
| R-squared | 0.38 |  |  | 0.46 |  |  | 0.37 |  |  | 0.41 |  |  |

Notes: significant threshold of 5%. Fixed-effects included surveyor and service providers dummies; interview conditions referred to the language of the interview and the attendance of the respondent.

When focusing on the subsample of individuals with intellectual disabilities, the association between QoL and living environment characteristics became less pronounced. Factors such as participation in day care or the type and location of residence did not show a significant effect on QoL. However, social interaction within the neighbourhood emerged as a key factor: individuals reporting less frequent contact with neighbours tended to experience lower levels of well-being (*β*=-0, 44, *p*=0.03; *β*=-0, 74, *p*=0.017; *β*=-0, 30, *p*=0.07). The objective needs for support remained a strong and statistically significant predictor of QoL in this subgroup (p = 0.004), highlighting the continued importance of support needs regardless of living arrangements. In the analysis restricted to self-respondents, the findings closely mirrored those of the full sample. Living environment variables and objective needs for support remained the primary determinants of QoL, suggesting that proxy responses did not substantially alter the overall conclusions.

Finally, we assessed the robustness of our findings by examining subsamples defined by different response rate thresholds on the QoL scale. Increasing the required response rate reduced the sample size (see Appendix S4, Table S4B ). Across these subsamples, objective needs for support, type of residence, and type of room consistently emerged as significant determinants of QoL. Participation in day care also showed a modest but noteworthy association. These results reinforce the reliability of the core findings across varying levels of data completeness. Participants excluded because of low response rates were more likely to have severe or profound intellectual disabilities and autism spectrum disorder. For example, among participants retained using the 60% response-rate threshold, 20% had severe or profound intellectual disabilities, compared with 14% among participants with a complete response rate (100%). Conversely, participants with moderate intellectual disabilities represented 21% of the sample at the 60% threshold, compared with 29% among those with complete responses. A similar pattern was observed for participants with autism spectrum disorder. No substantial differences were observed in terms of gender composition across the different response-rate thresholds. However, some differences emerged with respect to age. Participants with lower response rates tended to be older: among individuals retained using the 60% threshold, 30% were aged 60 years or older, compared with 25% among participants with complete responses.

Table S4B. Response rate and sample size

| QoL scale - response rate (%) | Sample size (n) |
| --- | --- |
| 100 | 73 |
| 90 | 97 |
| 80 | 108 |
| 70 | 115 |
| 60 | 124 |
| 50 | 127 |
| 40 | 128 |
| 30 | 131 |
| 20 | 131 |
| 10 | 132 |
| 0 | 139 |

Notes: the raw sample was composed of 152 respondents. 13 minors were excluded.
