# Supplementary material for: Factors associated with the quality of life of people with disabilities living in institutions or attending day care in Luxembourg
Source: PLoS One. 2026 Sep 8;21(9):e0356610. doi: 10.1371/journal.pone.0356610 (PMC13552743; doi:10.1371/journal.pone.0356610)
Supplement: Supplementary file 5 [file pone.0356610.s006.docx]

Dear reviewer,

Thank you for your thorough and insightful review of our manuscript. We greatly appreciate your insightful comments and suggestions, which have helped us improve the manuscript. Our point-by-point responses to your comments are provided below.

__________________________________________________________________________________

**Reviewer #2**

This topic is important and policy relevant. The strength of this manuscript being author has focused on environmental and social participation factors in addition to disability characteristics. Author has l done a good job.
However, there are few suggestion and questions to the author:
1.Title: The title and abstract use the word “determinant” which usually implies a casual relationship. This study is cross sectional, so term like “correlates” or “association “or “factors associated with” would be more methodological accurate.

**Following the reviewer’s suggestion, we have revised the title to “Factors associated with the quality of Life of people with disabilities living in institutions or attending day care in Luxembourg”.**

2.Proxy vs self-report respondents: Some interview involved proxies or third-party respondents. In disability research proxy reports frequently differs from self-report, especially regarding internal state of a person (quality of life), because QoL is intrinsically subjective. Can author clarify on?

a. How many interviews involved proxy participants?

**There were seven interviews (5%) involving proxy participants.**

b. Whether these cases differed from self-respondent?

**We acknowledge that self-respondents and proxy respondents may differ in terms of QoL as this is an inherently subjective construct that may be over- or under-reported by a trusted proxy. The average QoL score in the full sample was slightly higher than that observed in the self-respondent subsample, suggesting that participants represented by a proxy reported, on average, lower QoL levels. These participants had severe or profound intellectual disabilities or autism spectrum disorder. However, it remains unclear whether this difference reflects genuinely lower QoL among proxy respondents or potential reporting bias associated with proxy assessments. We therefore conducted sensitivity analyses, as suggested by the reviewer, to assess the robustness of our findings (p.16).**

c. Whether sensitivity analyses were done excluding proxy cases?

**We have now addressed this issue by conducting a sensitivity analysis using the subsample of self-respondents (Table S4A in Appendix S4). The self-respondent sample comprised 117 participants. The factors associated with quality of life were largely consistent with the benchmark results, with the living environment domain showing comparable associations.**

d.Whether proxy answered fully or partially?

**To improve clarity, we now provide a more detailed description of proxy respondents in the manuscript and explain the circumstances under which proxy interviews were conducted. We have revised the manuscript in three places to clarify the relationship between face-to-face interviews and proxy interviews. While all interviews were conducted face-to-face in the sense that an interviewer was physically present, in some cases, the participant was not present or was unable to engage in the interview situation due to communication or behavioural reasons. In such cases, a trusted person responded entirely on their behalf, which we refer to as a proxy interview.**

**This is now explicitly defined in:**

- **the Methods section (Procedure, p. 6, l.145-147): “When the participant was not present, a trusted person responded entirely on their behalf (proxy interview). The proxy interviewer was expected to have known the disabled person for at least one year in typical daily situations.”**
- **repeated in the Robustness section (p. 16, l.303-305): “Second, in some cases, the respondent did not personally participate in the interview and was replaced by a proxy, a trusted person who responded on their behalf.”**
- **and further contextualised in the Discussion (p. 19, l.411-413): “As a result, people in our sample may have more difficulties in communicating or expressing themselves and therefore require the presence of a proxy, a trusted person who responded on their behalf.”, where we acknowledge that participants in our sample may have more difficulties in communicating or expressing themselves, or may not be able to engage in an interview situation, and therefore require the presence of a proxy.**

3.Table 5 contains substantial information but is difficult to interpret quickly. If possible, author can simplify the presentation in table, highlighting the main finding.

**Table 5 has been revised, and results that are statistically significant at the conventional 1% and 5% levels are now presented in bold for ease of interpretation.**

4.The manuscript uses an adapted quality of life scale derived from Schlock’s framework. As per manuscript two items were excluded after calculation of alpha. Can you clarify?

**We used an adapted quality of life scale derived from Schalock's framework. To assess the internal consistency of the scale, we calculated Cronbach's alpha coefficient. Cronbach's alpha is a widely used measure of reliability that evaluates the extent to which items intended to measure the same underlying construct are correlated with one another. A higher value indicates greater consistency among the items and therefore stronger reliability of the scale. The analysis indicated that two items exhibited low item-total correlations and were not sufficiently consistent with the underlying construct measured by the scale. Consequently, these items were removed from the final analysis to improve the scale's internal consistency. These two items were “My life is very stressful” and “What is important to me in my life is often not respected/supported” (Table 1, p.6). We have amended the relevant section of the manuscript to clarify the role of Cronbach's alpha and to explicitly identify the two items that were excluded from the analysis.**

a. Why this shortened scale chosen?

**The main reason for using a shorter version of the instrument was to limit the overall administration time of the questionnaire. Given the characteristics of the study population, a lengthy questionnaire could have increased respondent burden, reduced attention, and raised the risk of participant dropout. We therefore opted for the shortened version of the Quality of Life questionnaire developed by Schalock et al. (1993), which allowed us to balance comprehensiveness with feasibility of administration.**

**The manuscript has been amended accordingly (p.6): “The QoL of individuals with disabilities was assessed using an adapted version of the widely recognized instrument developed by Schalock et al. (1993). This model conceptualizes QoL across eight key domains: emotional well-being, interpersonal relations, material well-being, personal development, physical well-being, self-determination, social inclusion, and rights. Guided by this framework, we constructed a 16-item scale designed to capture a broad yet concise representation of these domains (see Table 1). In adapting Schalock’s instrument, we did not include the full original questionnaire. Given its length, the shorter version was selected to reduce respondent burden, maintain participant engagement throughout the interview, and minimize the risk of fatigue, incomplete responses, and dropout. The questionnaire also encompassed other important constructs, such as practicability, acceptability, and temporal burden, which required limiting the number of items to prevent respondent fatigue. The 16 items included in the QoL measure were carefully selected to ensure balanced coverage of the eight domains, while maintaining alignment with Schalock’s theoretical model (Table 1). The respondents answered using a 5-point Likert scale, from 1 (completely true) to 5 (not true at all). When needed, the items were recoded, so that the higher the score, the greater the QoL.”**

b. Whether any factor structure was explored?
It would be better if manuscript explain about it.

**We did not undertake any factor analysis.**

5.Regarding Raw sample size and response rate: Table D2 shows that raw sample size was 152, but only 124 were analyzed. Similarly same table shows that only 73 individuals completed 100% of QoL scale.
Can author provide the details of those dropped out?

**The initial sample consisted of 152 participants, including both adults and minors. As the present study focuses on adults, 13 participants under the age of 18 were excluded, resulting in a sample of 139 adult participants.**

**We then considered the response rate for the dependent variable, QoL. It was measured using a scale composed of 14 items, each corresponding to a statement presented to participants (Table 1, p.6). To ensure the validity and reliability of the individual QoL scores, we restricted the analysis to participants who completed the majority of the items. Including individuals who responded to only a small number of statements could lead to QoL scores that do not adequately reflect the underlying construct and are therefore of limited validity.**

**Among the 139 adult participants, 124 completed at least 60% of the QoL items and were therefore retained for the main analysis. We selected this threshold as a compromise between maximizing sample size and ensuring sufficient information to construct a meaningful QoL score. Some participants exhibited lower response rates because they experienced difficulties with communication, concentration, or sustained engagement during the interview, while others simply chose not to answer certain items.**

**This is now explicitly explained and the manuscript has been amended accordingly (Appendix D, l.861): “Participants excluded because of low response rates were more likely to have severe or profound intellectual disabilities and autism spectrum disorder. For example, among participants retained using the 60% response-rate threshold, 20% had severe or profound intellectual disabilities, compared with 14% among participants with a complete response rate (100%). Conversely, participants with moderate intellectual disabilities represented 21% of the sample at the 60% threshold, compared with 29% among those with complete responses. A similar pattern was observed for participants with autism spectrum disorder. No substantial differences were observed in terms of gender composition across the different response-rate thresholds. However, some differences emerged with respect to age. Participants with lower response rates tended to be older: among individuals retained using the 60% threshold, 30% were aged 60 years or older, compared with 25% among participants with complete responses.”**

**To assess the robustness of our findings, we also conducted sensitivity analyses using alternative response-rate thresholds of 50% and 70%. As expected, applying a more stringent threshold reduced the sample size and limited the feasibility of some statistical analyses. Nevertheless, the results remained broadly consistent with the main findings, suggesting that our conclusions are not sensitive to the choice of response-rate threshold. Although a sample restricted to participants with complete responses would be ideal from a methodological perspective, such a criterion would substantially reduce the sample size and compromise statistical power.**

6. The authors concluded that disability type, communication skills and mobility were not significantly associated with QoL. The absence of statistical significance does not prove absence of association.

**We agree that non-significant coefficients should not be interpreted as evidence that disability type, communication skills or mobility have no association with QoL. Our intention was to state that, in this sample and in the adjusted models, we did not detect statistically significant independent associations for these variables.**

**We have therefore revised the Results, Abstract and Discussion to use more cautious language. In particular, we now avoid statements such as “did not influence QoL” or “disability itself does not affect QoL”. Instead, we refer to “no statistically significant association detected in the present sample” and emphasize that the estimates should be interpreted with caution, particularly given the modest sample size and the small number of participants in some disability subgroups, including autism and poly-disability.**
