# Supplementary material for: Factors associated with the quality of life of people with disabilities living in institutions or attending day care in Luxembourg
Source: PLoS One. 2026 Sep 8;21(9):e0356610. doi: 10.1371/journal.pone.0356610 (PMC13552743; doi:10.1371/journal.pone.0356610)
Supplement: Peer review history

# Peer review: Decision Letter 0

**DOI:** 10.1371/journal.pone.0356610.r001

**Authors:** Saraswati Dhungana

12 May 2026

Determinants of the Quality of Life of People with Disabilities living in Institutions or attending Day Care in Luxembourg

PLOS One

Dear Dr.  Blaise,

Thank you for submitting your manuscript to PLOS ONE. After careful consideration, we feel that it has merit but does not fully meet PLOS ONE’s publication criteria as it currently stands. Therefore, we invite you to submit a revised version of the manuscript that addresses the points raised during the review process.

Please submit your revised manuscript by Jun 26 2026 11:59PM. If you will need more time than this to complete your revisions, please reply to this message or contact the journal office at plosone@plos.org. When you're ready to submit your revision, log on to https://www.editorialmanager.com/pone/ and select the 'Submissions Needing Revision' folder to locate your manuscript file.

- A letter that responds to each point raised by the academic editor and reviewer(s). You should upload this letter as a separate file labeled 'Response to Reviewers'.
- A marked-up copy of your manuscript that highlights changes made to the original version. You should upload this as a separate file labeled 'Revised Manuscript with Track Changes'.
- An unmarked version of your revised paper without tracked changes. You should upload this as a separate file labeled 'Manuscript'.

If you would like to make changes to your financial disclosure, please include your updated statement in your cover letter. Guidelines for resubmitting your figure files are available below the reviewer comments at the end of this letter.

If applicable, we recommend that you deposit your laboratory protocols in protocols.io to enhance the reproducibility of your results. Protocols.io assigns your protocol its own identifier (DOI) so that it can be cited independently in the future. For instructions see: https://journals.plos.org/plosone/s/submission-guidelines#loc-laboratory-protocols. Additionally, PLOS ONE offers an option for publishing peer-reviewed Lab Protocol articles, which describe protocols hosted on protocols.io. Read more information on sharing protocols at https://plos.org/protocols?utm_medium=editorial-email&utm_source=authorletters&utm_campaign=protocols.

As the corresponding author, your ORCID iD is verified in the submission system and will appear in the published article. PLOS supports the use of ORCID, and we encourage all coauthors to register for an ORCID iD and use it as well. Please encourage your coauthors to verify their ORCID iD within the submission system before final acceptance, as unverified ORCID iDs will not appear in the published article. *Only*  the individual author can complete the verification step; PLOS staff *cannot*  verify ORCID iDs on behalf of authors.

We look forward to receiving your revised manuscript.

Kind regards,

Saraswati Dhungana, MD, PhD

Academic Editor

PLOS One

Journal Requirements:

When submitting your revision, we need you to address these additional requirements.

1.Please ensure that your manuscript meets PLOS ONE’s style requirements, including those for file naming. The PLOS ONE style templates can be found at

https://journals.plos.org/plosone/s/file?id=wjVg/PLOSOne_formatting_sample_main_body.pdf and

https://journals.plos.org/plosone/s/file?id=ba62/PLOSOne_formatting_sample_title_authors_affiliations.pdf

2. We note that the grant information you provided in the ‘Funding Information’ and ‘Financial Disclosure’ sections do not match.

When you resubmit, please ensure that you provide the correct grant numbers for the awards you received for your study in the ‘Funding Information’ section.

3. Thank you for stating the following financial disclosure:

“This work was supported by the Luxembourg Ministry of Family Affairs, Solidarity, Living Together and Reception of Refugees.”

Please state what role the funders took in the study.  If the funders had no role, please state: "The funders had no role in study design, data collection and analysis, decision to publish, or preparation of the manuscript."

If this statement is not correct you must amend it as needed.

Please include this amended Role of Funder statement in your cover letter; we will change the online submission form on your behalf.

4. We note that you have indicated that there are restrictions to data sharing for this study. PLOS only allows data to be available upon request if there are legal or ethical restrictions on sharing data publicly. For more information on unacceptable data access restrictions, please see http://journals.plos.org/plosone/s/data-availability#loc-unacceptable-data-access-restrictions.

Before we proceed with your manuscript, please address the following prompts:

a) If there are ethical or legal restrictions on sharing a de-identified data set, please explain them in detail (e.g., data contain potentially identifying or sensitive patient information, data are owned by a third-party organization, etc.) and who has imposed them (e.g., a Research Ethics Committee or Institutional Review Board, etc.). Please also provide contact information for a data access committee, ethics committee, or other institutional body to which data requests may be sent.

b) If there are no restrictions, please upload the minimal anonymized data set necessary to replicate your study findings to a stable, public repository and provide us with the relevant URLs, DOIs, or accession numbers. For a list of recommended repositories, please see

https://journals.plos.org/plosone/s/recommended-repositories. You also have the option of uploading the data as Supporting Information files, but we would recommend depositing data directly to a data repository if possible.

We will update your Data Availability statement on your behalf to reflect the information you provide.

5. Please include captions for your Supporting Information files at the end of your manuscript, and update any in-text citations to match accordingly. Please see our Supporting Information guidelines for more information: http://journals.plos.org/plosone/s/supporting-information.

6. If the reviewer comments include a recommendation to cite specific previously published works, please review and evaluate these publications to determine whether they are relevant and should be cited. There is no requirement to cite these works unless the editor has indicated otherwise.

Additional Editor Comments:

This is an interesting paper where the authors have attempted to explore the association of various factors in determining quality of life in people with disabilities in Luxembourg in a holistic manner. Overall, the paper gives valuable insights into the relation stating that quality of life in people with disabilities should be studied from a different lens as opposed to studying from medical model only.

I have some comments in regard to the manuscript, mainly in the methodology section and I would like the authors to clarify these.

One of the rationales for the study the authors gave was inclusion of all forms of disabilities, unlike in other studies, however, I do not see visual and hearing disabilities, which are quite common. Is there any specific reason for not including these?

Were there clear inclusion and exclusion criteria? What is the sampling process? The authors have stated that the sampling frame comprised 1115 people and they applied stratified sampling to derive the sample size of 152 based on 4 criteria. Please clarify the process.

The authors are advised to clarify what active and passive participation meant in this context? Besides, there is mention of face-to-face interviews being carried for all the participants. I was wondering what this meant when the authors also mention about proxy interviews. Furthermore, for those with no verbal fluency and even for those with verbal fluency but low intelligence, how did the authors validate the responses in the QoL scale.

The authors state that the questions were shortened and only required number of items were included to avoid respondent fatigue. While this sounds logical, I was wondering if the scale allowed such modification. The authors could be more explicit on the tool, its psychometric properties and the items in the original and the modified tool. Did the authors consult with the original developers regarding the modification and if this was permitted and if it introduced any form of bias. Which were the items that were omitted and why? On what basis were the particular items chosen? There are various items which require critical thinking, for instance, Item 7: I was considered a stranger. This is a very vague statement with various meanings. Do you think the participants understood all the items in the scale? The authors are advised to comment on this statement and if it affects the validity of the tool.

The authors mention about pilot interviews. Please elaborate on this. What do you mean? On whom and why? Any modification made thereafter and why were these needed?

Besides, the discussion section needs further rewriting with focus on QoL in context of disability in Luxembourg and elsewhere.

[Note: HTML markup is below. Please do not edit.]

Reviewer’s Responses to Questions

**Comments to the Author**

1. Is the manuscript technically sound, and do the data support the conclusions?

Reviewer #1: Yes

Reviewer #2: Partly

**********

2. Has the statistical analysis been performed appropriately and rigorously?

Reviewer #1: Yes

Reviewer #2: I Don't Know

**********

3. Have the authors made all data underlying the findings in their manuscript fully available?

The PLOS Data policy

Reviewer #1: Yes

Reviewer #2: Yes

**********

4. Is the manuscript presented in an intelligible fashion and written in standard English?

Reviewer #1: Yes

Reviewer #2: Yes

**********

Reviewer #1: Sampling technique not mentioned in abstract

Reason for selection of Quality Of Life tool not mentioned

Discussion:

Survey done during COVID and its impact on correlation between quality of life and social/neighborhood interaction found in the study. Need to Emphasize on question on social interactions were based on condition before COVID restrictions in methodology not only in appendix.

Limitations:

Low number of participants with autism and polydisability

Reviewer #2: This topic is important and policy relevant. The strength of this manuscript being author has focused on environmental and social participation factors in addition to disability characteristics. Author has l done a good job.

However, there are few suggestion and questions to the author:

1.Title: The title and abstract use the word “determinant” which usually implies a casual relationship. This study is cross sectional, so term like “correlates” or “association “or “factors associated with” would be more methodological accurate.

2.Proxy vs self-report respondents: Some interview involved proxies or third-party respondents. In disability research proxy reports frequently differs from self-report, especially regarding internal state of a person (quality of life), because QoL is intrinsically subjective.

Can author clarify on?

a. How many interviews involved proxy participants?

b. Whether these cases differed from self-respondent?

c. Whether sensitivity analyses were done excluding proxy cases?

d.Whether proxy answered fully or partially?

3.Table 5 contains substantial information but is difficult to interpret quickly. If possible, author can simplify the presentation in table, highlighting the main finding.

4.The manuscript uses an adapted quality of life scale derived from Schlock’s framework. As per manuscript two items were excluded after calculation of alpha. Can you clarify?

a. Why this shortened scale chosen?

b. Whether any factor structure was explored?

It would be better if manuscript explain about it.

5.Regarding Raw sample size and response rate: Table D2 shows that raw sample size was 152, but only 124 were analyzed. Similarly same table shows that only 73 individuals completed 100% of QoL scale.

Can author provide the details of those dropped out?

6. The authors concluded that disability type, communication skills and mobility were not significantly associated with QoL. The absence of statistical significance does not prove absence of association.

**********

what does this mean?). If published, this will include your full peer review and any attached files.

If you choose “no”, your identity will remain anonymous but your review may still be made public.

**Do you want your identity to be public for this peer review?**  For information about this choice, including consent withdrawal, please see our Privacy Policy

Reviewer #1: No

Reviewer #2: **Yes:**  Prof.Dr.Manisha Chapagai

**********

[NOTE: If reviewer comments were submitted as an attachment file, they will be attached to this email and accessible via the submission site. Please log into your account, locate the manuscript record, and check for the action link "View Attachments". If this link does not appear, there are no attachment files.]

To ensure your figures meet our technical requirements, please review our figure guidelines: https://journals.plos.org/plosone/s/figures

You may also use PLOS’s free figure tool, NAAS, to help you prepare publication quality figures: https://journals.plos.org/plosone/s/figures#loc-tools-for-figure-preparation.

NAAS will assess whether your figures meet our technical requirements by comparing each figure against our figure specifications.

# Author comment: Author response to Decision Letter 1

**DOI:** 10.1371/journal.pone.0356610.r002

8 Jul 2026

Dear Editor,

Dear reviewers,

We sincerely thank you for your time and constructive feedback throughout the review process. Your suggestions have helped us improve the quality and clarity of the manuscript. Please find our detailed responses in the attached documents.

Best regards,

Attachment Submitted filename: Comments_R2.docx

# Peer review: Decision Letter 1

**DOI:** 10.1371/journal.pone.0356610.r003

**Authors:** Saraswati Dhungana

5 Aug 2026

Factors associated with the quality of life of people with disabilities living in institutions or attending day care in Luxembourg

PONE-D-26-11757R1

Dear Dr. Blaise,

We’re pleased to inform you that your manuscript has been judged scientifically suitable for publication and will be formally accepted for publication once it meets all outstanding technical requirements.

Within one week, you’ll receive an e-mail detailing the required amendments. When these have been addressed, you’ll receive a formal acceptance letter and your manuscript will be scheduled for publication.

An invoice will be generated when your article is formally accepted. Please note, if your institution has a publishing partnership with PLOS and your article meets the relevant criteria, all or part of your publication costs will be covered. Please make sure your user information is up-to-date by logging into Editorial Manager at Editorial Manager® and clicking the ‘Update My Information' link at the top of the page. For questions related to billing, please contact billing support.

If your institution or institutions have a press office, please notify them about your upcoming paper to help maximize its impact. If they’ll be preparing press materials, please inform our press team as soon as possible -- no later than 48 hours after receiving the formal acceptance. Your manuscript will remain under strict press embargo until 2 pm Eastern Time on the date of publication. For more information, please contact onepress@plos.org.

Kind regards,

Saraswati Dhungana, MD

Academic Editor

PLOS One

Additional Editor Comments (optional):

Reviewers' comments:

Reviewer’s Responses to Questions

**Comments to the Author**

Reviewer #2: All comments have been addressed

**********

2. Is the manuscript technically sound, and do the data support the conclusions?

Reviewer #2: Yes

**********

3. Has the statistical analysis been performed appropriately and rigorously?

Reviewer #2: Yes

**********

4. Have the authors made all data underlying the findings in their manuscript fully available?

The PLOS Data policy

Reviewer #2: Yes

**********

5. Is the manuscript presented in an intelligible fashion and written in standard English?

Reviewer #2: Yes

**********

Reviewer #2: All my queries have been satisfactorily addressed. I have no further comments . From my side manuscript looks ok .

Thank you

**********

what does this mean?). If published, this will include your full peer review and any attached files.

If you choose “no”, your identity will remain anonymous but your review may still be made public.

**Do you want your identity to be public for this peer review?**  For information about this choice, including consent withdrawal, please see our Privacy Policy

Reviewer #2: **Yes:** Dr. Manisha Chapagai

**********

# Editor report: Acceptance letter

**DOI:** 10.1371/journal.pone.0356610.r004

**Authors:** Saraswati Dhungana

PONE-D-26-11757R1

PLOS One

Dear Dr. Blaise,

I'm pleased to inform you that your manuscript has been deemed suitable for publication in PLOS One. Congratulations! Your manuscript is now being handed over to our production team.

At this stage, our production department will prepare your paper for publication. This includes ensuring the following:

* All references, tables, and figures are properly cited

* All relevant supporting information is included in the manuscript submission,

* There are no issues that prevent the paper from being properly typeset

You will receive further instructions from the production team, including instructions on how to review your proof when it is ready. Please keep in mind that we are working through a large volume of accepted articles, so please give us a few days to review your paper and let you know the next and final steps.

Lastly, if your institution or institutions have a press office, please let them know about your upcoming paper now to help maximize its impact. If they'll be preparing press materials, please inform our press team within the next 48 hours. Your manuscript will remain under strict press embargo until 2 pm Eastern Time on the date of publication. For more information, please contact onepress@plos.org.

You will receive an invoice from PLOS for your publication fee after your manuscript has reached the completed accept phase. If you receive an email requesting payment before acceptance or for any other service, this may be a phishing scheme. Learn how to identify phishing emails and protect your accounts at https://explore.plos.org/phishing.

If we can help with anything else, please email us at customercare@plos.org.

Thank you for submitting your work to PLOS One and supporting open access.

Kind regards,

PLOS One Editorial Office Staff

on behalf of

Dr. Saraswati Dhungana

Academic Editor

PLOS One
